# The Standing Pool of Genomic Structural Variation in a Natural Population of *Mimulus guttatus*

**DOI:** 10.1093/gbe/evt199

**Published:** 2013-12-12

**Authors:** Lex E. Flagel, John H. Willis, Todd J. Vision

**Affiliations:** ^1^Department of Biology, Duke University; ^2^Department of Biology, University of North Carolina, Chapel Hill; ^3^Present address: Monsanto Company, Chesterfield, MO

**Keywords:** indel, *Mimulus guttatus*, natural selection, population genomics, structural variation

## Abstract

Major unresolved questions in evolutionary genetics include determining the contributions of different mutational sources to the total pool of genetic variation in a species, and understanding how these different forms of genetic variation interact with natural selection. Recent work has shown that structural variants (SVs) (insertions, deletions, inversions, and transpositions) are a major source of genetic variation, often outnumbering single nucleotide variants in terms of total bases affected. Despite the near ubiquity of SVs, major questions about their interaction with natural selection remain. For example, how does the allele frequency spectrum of SVs differ when compared with single nucleotide variants? How often do SVs affect genes, and what are the consequences? To begin to address these questions, we have systematically identified and characterized a large set of submicroscopic insertion and deletion (indel) variants (between 1 and 200 kb in length) among ten inbred lines from a single natural population of the plant species *Mimulus guttatus*. After extensive computational filtering, we focused on a set of 4,142 high-confidence indels that showed an experimental validation rate of 73%. All but one of these indels were less than 200 kb. Although the largest were generally at lower frequencies in the population, a surprising number of large indels are at intermediate frequencies. Although indels overlapping with genes were much rarer than expected by chance, approximately 600 genes were affected by an indel. Nucleotide-binding site leucine-rich repeat (NBS–LRR) defense response genes were the most enriched among the gene families affected. Most indels associated with genes were rare and appeared to be under purifying selection, though we do find four high-frequency derived insertion alleles that show signatures of recent positive selection.

## Introduction

From comparative genomics, we know that a large portion of the genetic differences between closely related species are structural in nature rather than substitutions involving single nucleotides (Britten 2002; Chimpanzee Sequencing and Analysis Consortium 2005; Hu et al. 2011). These structural variants (SVs) include insertions, deletions, inversions, and transpositions. Many SVs are now fixed within species, but must have arisen and increased in frequency for some period of time prior to reaching fixation. From this, we might anticipate there to be a significant pool of standing genetic variation for SVs within species or even within populations. In the past, SVs could only be studied if they were large enough to have a visible manifestation, such as the inversions seen in *Drosophila* salivary chromosomes (Sturtevant and Dobzhansky 1936). With the advent of new technologies, we now have an unprecedented ability to discover much smaller “submicroscopic” SVs in a systematic manner on a genome-wide scale. With these tools, we can begin to address the contribution of SVs to the total pool of genetic variation in a population and determine how these polymorphisms interact with natural selection.

Various genomic technologies have allowed researchers to detect and catalog submicroscopic SVs (Feuk et al. 2006). The first wave of techniques involved microarrays specifically built to interrogate the genome with oligonucleotide probes (Borevitz et al. 2003). Recent approaches have capitalized on next-generation sequencing as a vehicle to generate millions of paired-end reads, which can be compared with a reference genome to discover SVs (Handsaker et al. 2011). These paired-end reads are small genomic fragments (∼500 bp) sequenced incompletely from both ends, creating a single-stranded 5′ to 3′ read on each strand with 200–300 bp of unsequenced insert in between. When comparing a newly sequenced accession to a reference genome, deviations from the expected insert size and expected read pair configuration can be used to identify SVs. Paired-end sequencing offers considerable improvements in SV resolution compared with earlier microarray-based technologies (Chen et al. 2009; Handsaker et al. 2011) and has the added benefit of also exposing single nucleotide polymorphisms (SNPs).

Studies of model organisms such as *Arabidopsis*, *Drosophila*, humans, and maize have revealed considerable levels of segregating structural polymorphism (Korbel et al. 2007; Emerson et al. 2008; Kidd et al. 2008; Swanson-Wagner et al. 2010; Cao et al. 2011; 1000 Genomes Project Consortium 2012; Chia et al. 2012). In the case of humans, the number of nucleotide differences between individuals due to SVs is reported to be greater than that due to SNPs (Redon et al. 2006). Although many of these studies have compared genotypes sampled from throughout the species’ range, recent studies in humans, three-spined stickleback, and *D. melanogaster* (Cridland and Thornton 2010; Mackay et al. 2012; Feulner et al. 2013; Montgomery et al. 2013) have shown that polymorphic SVs also contribute to the standing genetic variation within populations.

Here, we report a population genomic analysis of a large sample of SVs segregating within a single well-studied natural population of the plant species *Mimulus guttatus.* The SVs were discovered through paired-end whole-genome shotgun sequencing of ten inbred lines from the focal population. We also sequenced two inbred lines from distantly related populations to determine the derived and ancestral allele for each polymorphism. We restrict our focus to large indels because we find that they are the class of submicroscopic SV that could be most reliably validated in silico. To provide a glimpse into the role of selection in shaping the population genomic diversity of large indels, we explore their spatial distribution in the genome, compare their allele frequency spectrum with that of SNPs, and test whether any large indels may be increasing in frequency due to direct or indirect or positive selection.

## Materials and Methods

### Plant Materials, DNA Extraction, and *M. guttatus* Reference Genome Resources

The plant materials used in this study are documented in table 1. Our focal population includes ten inbred lines extracted from a natural population on Iron Mountain, OR, USA. Nine were chosen at random from a pool of 257 inbred lines, while the tenth, IM62, was chosen because it was also used to create the reference genome. In addition to this focal population, we chose two inbred lines extracted from distant populations (DUN and SF5). All plants were grown in the Duke University Biology Greenhouse. Leaf and bud tissues were harvested for DNA extraction when plants began to flower. DNA was extracted following a urea extraction protocol modified from Shure et al. (1983). All accessions used in this study were inbred through self-fertilization and single-seed descent at least six generations prior to DNA extraction and sequencing.

**Table 1:** Resequenced Accessions, Including Outgroups

Line	Species	Origin^a^	Generations of Inbreeding	Seq. Facility^b^	Total Paired End Reads	Read Type (bp)	Sites Available	Median Per Site Coverage^c^	NCBI SRA Accession No.
IM109	*M. guttatus*	Iron Mountain	11	UNC	24,671,221	2 × 75	127,863,968	8	SRX021073
IM1145	*M. guttatus*	Iron Mountain	11	UNC	22,839,207	2 × 75	138,331,815	8	SRX021074
IM155	*M. guttatus*	Iron Mountain	12	Duke	37,172,361	2 × 75	138,971,514	15	SRX055301
IM320	*M. guttatus*	Iron Mountain	15	Duke	23,226,015	2 × 75	160,689,132	8	SRX055300
IM479	*M. guttatus*	Iron Mountain	9	UNC	24,086,031	2 × 75	134,867,795	9	SRX021077
IM62	*M. guttatus*	Iron Mountain	>10	UNC	24,911,877	2 × 75	206,733,050	7	SRX021072
IM624	*M. guttatus*	Iron Mountain	13	UNC	22,433,144	2 × 75	137,605,733	8	SRX021075
IM693	*M. guttatus*	Iron Mountain	9	UNC	21,969,210	2 × 75	133,128,765	8	SRX021078
IM767	*M. guttatus*	Iron Mountain	11	UNC	25,497,466	2 × 75	135,649,759	9	SRX021079
IM835	*M. guttatus*	Iron Mountain	13	UNC	17,966,309	2 × 75	129,433,596	6	SRX021076
DUN	*M. guttatus*	Florence	>6	JGI	262,093,335	2 × 35	94,024,553	23	SRX030973, SRX030974
SF5	*M. nasutus*	Sherar’s Falls	Natural selfer	JGI	24,199,117	2 × 76	65,812,268	10	SRX116529

^a^All accessions originate from Oregon, USA. Approximate geographic coordinates as follows: Iron Mountain [44.4005, −122.1428], Florence [43.8891, −124.1360], and Sherar’s Falls [45.2587, −121.0201], with [Latitude, Longitude] given in decimal format.

^b^Duke University Sequence Facility (Duke), DOE Joint Genome Institute (JGI), University of North Carolina High-Throughput Sequencing Facility (UNC).

^c^Nucleotide sites belonging to reads with mapping quality scores ≥ 29.

We used the *M. guttatus* version 2.0 genome assembly (ftp://ftp.jgi-psf.org/pub/compgen/phytozome/v9.0/early_release/Mguttatus_v2.0/, last accessed December 16, 2013) with the version 1.1 annotation of genes and transposable elements that were available at the time (ftp://ftp.jgi-psf.org/pub/compgen/phytozome/v9.0/Mguttatus/annotation/, last accessed December 16, 2013). Coordinate mappings between assembly versions are available at the Dryad digital repository (http://dx.doi.org/10.5061/dryad.41dq8).

### Sequencing

DNA samples were sent to the DOE Joint Genome Institute, Duke University Sequence Facility, and the University of North Carolina High-Throughput Sequencing Facility (table 1), for library preparation with the Illumina Paired-end Sample Prep. Kit V1, followed by Illumina GAII sequencing. Sequence output is available in table 1, as are the NCBI-SRA accession numbers for all raw sequence data. Paired-end sequencing was performed in 2 × 35, 2 × 75, or 2 × 76 bp configurations. The mean distance between paired-end reads for all libraries was 275.8 bp and the mean within library standard deviation was ±28.8 bp.

### Alignment to Reference Genome and Identification of SNPs and Abnormally Aligned Read Pairs

Sequences were aligned to the *M. guttatus* reference genome using the Burrows–Wheeler Aligner (BWA version 0.5.8c [Li and Durbin 2010]), with all settings left at defaults, and utilizing the paired-end read alignment option (*sampe*).

### Identifying SNPs

SNPs were determined using the *pileup* function in the samtools package (version 0.1.8; [Li et al. 2009]). First we extracted all read pairs with BWA mapping quality ≥29 and then identified sites with at least 3× coverage but no greater than 25× coverage, except for the DUN accession, which we allowed a maximum coverage of 40×. From these sites, we made a base call if more than 75% of the reads displayed the same nucleotide. Finally, we only called SNPs in sites that passed these criteria for all ten Iron Mountain accessions. All SNPs in coding regions were assigned as synonymous or nonsynonymous using the coding frame of the longest predicted transcript at that locus in the *M. guttatus* Phytozome v9.0 genome annotation. All SNP calls can be found in the Supplemental SNP data set at the Dryad digital repository (http://dx.doi.org/10.5061/dryad.41dq8).

### Identifying Abnormally Aligned Read Pairs

Using samtools (version 0.1.8 [Li et al. 2009]) to traverse the alignments, we identified all read pairs for which both members align to the *M. guttatus* reference genome with a mapping quality ≥29, but have abnormal relative alignment positions (pairs not in the expected orientation (→←) and/or an insert size ≥1,000 bp). This information was assessed using information encoded in the bitwise SAM file flag values (supplementary table S3, Supplementary Material online). Among all lines, we identified 527,059 read pairs with abnormal alignments, and these were retained for further examination.

Next, as a means of minimizing alignment errors, all abnormally aligned read pairs were realigned to the reference genome with novoalign (version 2.07.11; http://www.novocraft.com, last accessed December 27, 2013) using a k-mer size of 14 (*k* = 14) and step size of 1 (*s* = 1), with all other parameters left at default settings. We chose novoalign, a hash-based aligner, because it uses a fundamentally different alignment algorithm than BWA, a Burrows–Wheeler transform based aligner. Novoalign identified novel high-quality (mapping quality ≥29) alignments that were not abnormal for 871 read pairs. After removing these read pairs, we were left with 526,188 abnormally aligned read pairs that had been confirmed by both BWA and novoalign.

### Clustering Abnormally Aligned Reads to Identify Putative SVs

Following the strategy of Chen et al. (2009), abnormally aligned reads from all accessions were pooled to make use of all available information when predicting SVs and localizing their breakpoints. After pooling, abnormally aligned read pairs were clustered into sets that came from the same genomic locations for both the forward and reverse read pairs. This clustering was done using the ClusterTree function in the bx-python package (version 0.7.0; http://pypi.python.org/pypi/bx-python, last accessed December 27, 2013), which provides a data structure for finding clusters of intervals where both endpoints fall within a certain window size. Based on the smallest mean insert size among our paired-end sequences, we chose 225 bp as our maximum window size. Furthermore, we also required that the putative SV clusters be supported by at least three read pairs, regardless of which accessions contributed those read pairs. All retained clusters were partitioned into SV classes (deletions, inversions, and transpositions) based on the paired read configuration. Finally, the accession(s) contributing to each cluster were assigned. Python code for read pair clustering is available at the Dryad digital repository (http://dx.doi.org/10.5061/dryad.41dq8).

### Filtering SVs

We resequenced the accession (IM62) that was used to construct the *M. guttatus* reference genome. By aligning paired-end reads from IM62 to itself, we were able to identify spurious SV calls and remove any SVs that included IM62 as one of the accessions containing the putative event. Also, we only retained SVs ≥ 1,000 bp. Because we were working with highly inbred lines, we expect nearly all loci to be homozygous, and accessions containing a predicted deletion relative to the IM62 reference genome should show few or no high-quality read alignments to the deleted interval. Following this principle, we only retained deletion events in which the putatively deleted interval had a read depth of coverage in the lowest 10th percentile of the genome-wide distribution for that accession. Also, we found that some regions in the *M. guttatus* genome produce a large number of abnormally aligned reads (supplementary fig. S1, Supplementary Material online). We suspect that repeats make these intervals difficult for read alignment, so we removed any SVs from these regions. This was done by counting the number of abnormally aligned reads for both endpoints of a candidate SV using a 5,000-bp window with the focal SV in the middle. After discounting all abnormally aligned reads assigned to the focal SV, we determined whether the count of additional abnormally aligned reads in this window was greater than the 90th percentile of all 5,000-bp windows in the genome. If it was, the focal SV was dropped. Also, for inversions and transpositions, we enforced that read coverage across the SV interval remain between the 10th and 90th percentile of the genome-wide distribution, to avoid regions that have either unusually sparse or dense coverage. Finally, if one accession failed the test when applying the filters listed above, the candidate SV was rejected for all accessions. Python code for filtering SVs is available from the Dryad digital repository (http://dx.doi.org/10.5061/dryad.41dq8). The cluster assignment, SV type, and filtering fate for all 527,059 abnormally aligned read pairs is available as a supplemental data set from the Dryad digital repository (http://dx.doi.org/10.5061/dryad.41dq8).

### Monte Carlo Methods, Coalescent Simulations, and Population Genetic Calculations

To obtain an estimate of the expected average absolute allele frequency difference for a sample size of ten individuals relative to the “true” allele frequency estimate from approximately 100 individuals, we performed a Monte Carlo simulation. We first drew a true allele frequency randomly from a uniform distribution between 0 and 1. We then randomly drew ten samples from a binomial distribution of size 1 using the true allele frequency as the probability of success. This sample makes up the simulated observed allele frequency from a sample size of ten, matching our sample size from the Iron Mountain population. Because our indel detection scheme was such that the minor allele had to be found in at least one accession, we censored the simulated observed values so they fell between 0.1 and 0.9. Finally, we collected the absolute difference between the true value and the censored observed value. This process was repeated 50,000 times, and the average of these replicates was 0.097.

The expected frequency spectrum for neutral loci was estimated using the standard neutral model as implemented by the ms coalescent simulation software package (Hudson 2002). We simulated 10,000 genealogies, each with ten unique haploid chromosomes (the effective number of genomes sampled from ten inbred accessions) and 100 segregating sites with no recombination. From these replicate genealogies, we calculated the average site frequency spectrum, the expected minor allele frequency (MAF) and the upper and lower bounds of the 95% confidence interval for Tajima’s *D* (−1.72, +1.59), and Normalized Fay and Wu’s *H* (−1.88, +5.70). Simulations indicated that the folded site frequency spectrum of synonymous variants was within the 95% confidence interval of the standard neutral model for all frequency classes except singletons, which were rarer than expected (results not shown). Thus, our lower confidence limits for Tajima’s *D* and Normalized Fay and Wu’s *H* will be conservative. Tajima’s *D* and Normalized Fay and Wu’s *H* (Zeng et al. 2006) were calculated using the EggLib package (version 2.1.5; De Mita and Siol 2012). Normalized Fay and Wu’s *H* requires specification of an ingroup and an outgroup. This was achieved by randomly selecting a value between one and five, and randomly assigning this many simulated chromosomes to the outgroup.

Intra-allelic nucleotide diversity (π_A_) was calculated for all synonymous and nonsynonymous SNPs and for genic deletion alleles. We focused on only the genic deletion alleles, as we expect that many of the insertion alleles are wild-type because they were predicted to maintain an open reading frame in the IM62 genome annotation. For each polymorphism type, we separated the two alleles and calculated π_A_ for each allele class on a 500 bp haplotype centered on the polymorphism. Singleton alleles (i.e., 10% frequency) were ignored because their π_A_ cannot be defined. π_A_ values were then aggregated by allele frequency and mean π_A_ at each frequency was used an estimate of allelic age. Diffusion equations used to transform π_A_ into estimates of the mean population-scaled coefficient of selection ($\bar{N_{e}S}$) are given in Kiezun et al. (2013).

### Availability of Data, Software, and Materials

Seeds from the inbred lines used in this study are available from the *Mimulus* Stock Center (www.mimulusevolution.org/stocks.php, last accessed January 3, 2014). Sequencing data have been deposited at the NCBI-SRA (table 1). Additional data and software are available at the Dryad digital repository (http://dx.doi.org/10.5061/dryad.41dq8).

## Results

### Identifying SVs from Paired-End Sequence Data

To identify candidate SVs, we obtained approximately 531 million paired-end sequences from 12 inbred accessions. Ten of these were derived from the focal population (Iron Mountain, OR) and two from outgroup populations (table 1). Nine of the individuals from the focal population were chosen at random from a collection of 257 viable inbreds extracted from the Iron Mountain population, while the tenth was the inbred accession used to create the reference genome.

The principal challenge in reliably detecting SVs from alignment of paired-end sequences against a reference is that chimeric read pairs and erroneous alignments generate a large number of false-positive SVs (Handsaker et al. 2011), and in fact many of the initial candidate SVs in our data set are physically impossible to reconcile with one another (supplementary fig. S1, Supplementary Material online). Thus, it was essential to filter SV predictions by exploiting additional signals in the data.

We used a progression of filtering steps, summarized later and described more fully in the Materials and Methods. The first filtering strategy was based on the data from resequencing the inbred accession used to create the reference genome. All abnormally aligned read pairs deriving from the reference accession, when aligned to itself, serve as a marker for regions of the genome that produce unreliable read alignments (supplementary fig. S1, Supplementary Material online).

Various classes of SV were further filtered based on expectations for patterns that would be seen in either false- or true-positives. For example, we removed putative indels that did not show the expected low read coverage within the putatively deleted interval. For inversions and transpositions, we removed any candidates that fell in genomic windows with elevated rates of abnormally aligned reads. These filters greatly reduced the number of putative SV calls under consideration. Of the initial set of 13,845 putative indels, 4,142 high-confidence indels were retained. For inversions, the reduction was from 141 putative events to 35. And for transpositions, the filtering steps reduced 716 putative events to just 3. The fate of all abnormally aligned reads and their attrition as a result of various filters is available as a supplementary data file at the Dryad digital repository (http://dx.doi.org/10.5061/dryad.41dq8).

### Validation by Polymerase Chain Reaction Assays

We then sought to experimentally validate a subset of the candidates passing these filters using polymerase chain reaction (PCR) assays that would yield different amplicons depending on which allele was present in the sample. For inversions and transpositions, many of the putative events derived from areas of the genome that consistently produced unreliable alignments. We attempted to validate seven of the retained inversions using PCR (those shown in supplementary fig. S2, Supplementary Material online), but were unable to develop unique primer pair combinations as all were located within regions rich in repetitive sequences. We did not attempt to validate any of the three transpositions. Although there probably are true inversions and transpositions among the accessions we resequenced, it remains a challenge to reliably identify them using paired-end reads. Our experience mirrors that described by the 1000 Genomes Project (1000 Genomes Project Consortium 2012), and, like these authors, here we focus our analyses on the high-confidence indels only.

We developed PCR assays to test the existence of 48 indel allele pairs. These assays were done using either one or two amplifications. For smaller indels, we used one PCR amplification external to the predicted indel boundary. A genotype homozygous for a deletion allele was expected to produce a small amplicon of an expected size, while a genotype with the insertion allele was expected to produce a longer reference length amplicon. For longer indels, it was not possible to amplify across the insertion allele so we used two amplifications, one with both primers external to the predicted indel boundaries, and one with one internal and one external primer. A genotype homozygous for a deletion allele is expected to produce an amplicon only with the external primer pair, whereas a genotype with an insertion allele is expected to produce a reference-length amplicon using only the internal/external primer combination. Among the 48 assayed indel predictions, 24 confirmed our in silico predictions, 9 showed evidence of a false-positive call, and 15 were inconclusive due to failed PCR amplification. Of the 33 conclusive assay results, the overall rate of validation was 72.7% (supplementary table S1, Supplementary Material online), a figure comparable with a recent study in humans (Montgomery et al. 2013). From these results, we conclude that the majority of high-confidence indels are true positives.

We note that our data set of indels does not include any insertions relative to the reference, because we only detect putative indels by having two read pairs aligned at positions separated by at least 1,000 bp in the reference genome (Materials and Methods).

### Population Genomics of Indel Alleles in the *Mimulus* Genome

To determine the accuracy of the allele frequencies observed in the initial sample of ten resequenced lines, we genotyped 14 of the validated indels in a larger number of inbred lines (average *N* = 128) extracted from the focal population (Iron Mountain). Assuming that allele frequencies in this large sample approximate the true population frequencies, we can estimate the error of the initial frequency estimates. The Pearson’s correlation coefficient between the frequency estimates was 0.83, and the slope of the relationship was 0.77. The average absolute difference between both estimates for all 14 indels was 0.126 (supplementary fig. S3, Supplementary Material online), which is only slightly higher than the expected value of 0.097 (Materials and Methods). Because the slope is near 1 and the average absolute difference is near its expected minima when accounting for sampling error, we conclude that an estimate of allele frequency from the initial sample can be roughly used as a proxy for population frequency.

Next, we checked to see whether the variable frequency of indels among the nine nonreference lines could be a result of variable sequencing depth. The number of indels observed and sequencing depth show little association, and in fact the Pearson’s correlation coefficient is slightly negative (*r* = −0.175; supplementary fig. S4, Supplementary Material online). The number of indel differences from the reference genome is better explained by the overall patterns of nucleotide differentiation among the lines, as evidenced by a positive correlation between SNP divergence from the reference genome and indel divergence from the reference genome (Pearson’s *r = *0.582; supplementary fig. S4, Supplementary Material online). From this, we conclude that we have sequenced at sufficient depth to detect low to intermediate frequency indel alleles.

**Fig. 1.—:**
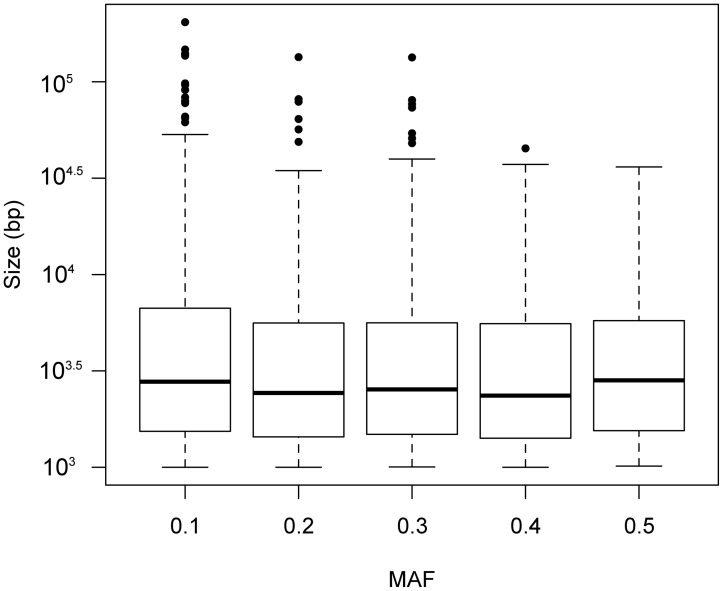
Distribution of indel size as a function of allele frequency. The box plots indicate the distribution of indel sizes (bp) at different MAF for all indels identified in the focal Iron Mountain population. Indel size (*y*-axis) is plotted on a log scale.

Indels in the Iron Mountain population ranged from 1,000 bp up to 204 kb with a median size of 2,562.5 bp (table 2). We asked whether large indels were disproportionately rare, as might be expected if larger indels are subject to stronger purifying selection. Because the ten accessions we resequenced were highly inbred (table 1), we observe only one allele per accession, and the MAF ranges from 0.1 to 0.5 by increments of 0.1. Within the Iron Mountain population, the mean MAF for all 4,142 indel polymorphisms was 0.255 (table 2). The mean indel size for each of the 5 MAF categories is as follows: 0.1 = 6,665 bp, 0.2 = 4,869 bp, 0.3 = 4,807 bp, 0.4 = 4,386 bp, and 0.5 = 4,307 bp. Indels with higher MAF tended to be smaller and have a more restricted upper size range when compared with indels with a low MAF (fig. 1), a result that is statistically significant (Welch’s one-way analysis of variance [ANOVA]; *F* [4, 1,829.5] = 7.33, *P* = 7.4 × 10^−^^6^). This observation suggests that purifying selection tends to be stronger for large indels, removing them from the population before they reach an appreciable allele frequency. Nonetheless, there exists a pool of large indels segregating at intermediate frequencies. For example, 9% of the indels with a MAF of 0.3 or higher are greater than 10 kb.

**Table 2:** Indel and SNP Variants among the Ten Resequenced Lines

Polymorphism Type	Count	Median Size (bp)	Average MAF^a^	Proportion of Derived Minor Alleles
Indels: all	4,142	2,563	0.255	0.718
Indels in genes	414	3,804	0.218	0.739
Indels in TEs	1,855	2,839	0.277	0.743
SNPs: all	1,337,759	1	0.222	0.619
SNPs: synonymous	259,676	1	0.239	0.550
SNPs: nonsynonymous	106,638	1	0.227	0.626

^a^Average MAF from neutral coalescent simulations = 0.222.

**Fig. 2.—:**
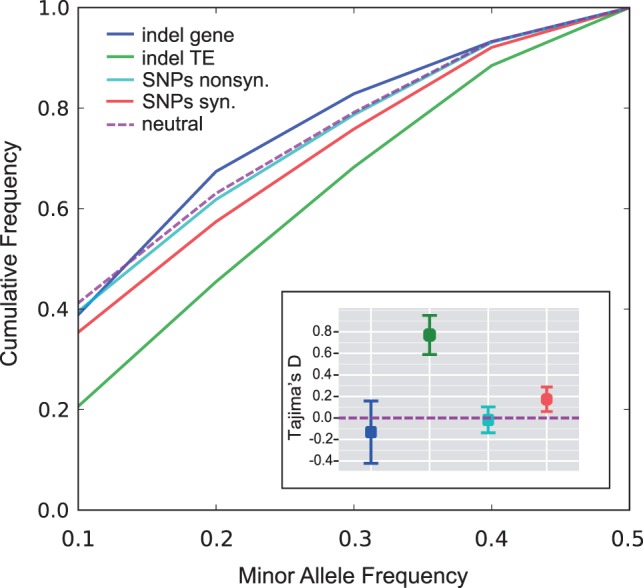
Allele frequency distributions in the ten resequenced lines. Cumulative frequency as a function of MAF is shown for genic and TE indels, synonymous and nonsynonymous SNPs, and a neutral coalescent simulation (neutral). Inset: Estimates of mean Tajima’s *D* color coded as in the main panel. Bars indicate the 95% confidence intervals as obtained by delete-one jackknifing the ten lines. Sample sizes are given in table 2.

To provide insight into how selective forces differ for large indels and SNPs, we compared their population genetic and genomic distributions. We extracted approximately 1.3 million SNPs from the resequencing data to compare the frequency and distribution of SVs and SNPs. The SNPs were partitioned into coding and noncoding polymorphisms and the coding SNPs were further partitioned into synonymous and nonsynonymous polymorphisms. The average MAF was similar for all classes of polymorphism, ranging from 0.218 to 0.277 (table 2). Figure 2 shows the cumulative allele frequency spectrum and Tajima’s *D* values for indels and SNPs. All values shown in figure 2 fall within the 95% confidence interval expected for polymorphisms experiencing neutral evolution as determined by coalescent simulations.

We used two distantly related individuals from outgroup populations (table 1) to polarize indels and SNPs. The minor allele for a large indel is more likely to be derived than it is for a SNP (0.718 for indels compared with 0.619 for SNPs; χ^2^ test of independence *P* = 4.2 × 10^−^^13^). This pattern could arise because indels have a higher mutation rate than SNPs, because purifying selection purges indels from the population faster than SNPs, or some combination of the two factors. We note that the SNP and indel polymorphisms examined here likely are less deleterious when homozygous than a sample drawn from nature. This is because the studied accessions were inbred by selfing for several generations prior to sequencing (table 1). By rapidly reducing heterozygosity, selfing exposed recessive mutations to atypically strong selection, likely purging many of the most deleterious alleles.

Across the *Mimulus* genome, there is considerable heterogeneity in the abundance of SNPs and large indels. To compare the distributions for the two types of polymorphism, we partitioned the genome into nonoverlapping 500 kb windows and tallied the segregating indels and SNPs in each window. To normalize for sequence coverage differences between windows, we divided the indel and SNP tallies by the total read coverage among all lines for each window. We then fit a linear model between the normalized indel and SNP counts in each window. We find a slight—albeit significant—positive relationship between the abundance of indels and SNPs in the genome (Pearson’s *r* = 0.208; slope = 4.9 × 10^−^^4^; df = 662; *P* = 6.3 × 10^−^^8^). However, SNP density explains only about 4% of the variation in indel density. This suggests that these mutational processes are weakly correlated throughout the genome, at least at the scale of 500 kb windows.

### Indels in Genes and TEs

There are approximately 27,000 genes and 239,000 TEs or TE fragments annotated in the *M. guttatus* genome, comprising 24.2% and 56.5% of the assembled nucleotides, respectively. We wanted to determine how indel polymorphisms were distributed with respect to these features. First, we isolated the alignable portions of the genome using nucleotide positions from the resequenced reference genome accession (IM62) with mapping qualities ≥29 as a guide (Materials and Methods). Following this filtering step, the alignable fraction was composed of 39.9% genes and 35.1% TEs, reflecting the relative uniqueness of gene sequences and the concentration of unalignable repetitive sequences among TEs (supplementary fig. S5, Supplementary Material online). The proportions from the alignable fraction of the genome represent the expected null genomic distribution of polymorphisms in our data after accounting for the bias of sequence alignment.

**Fig. 3.—:**
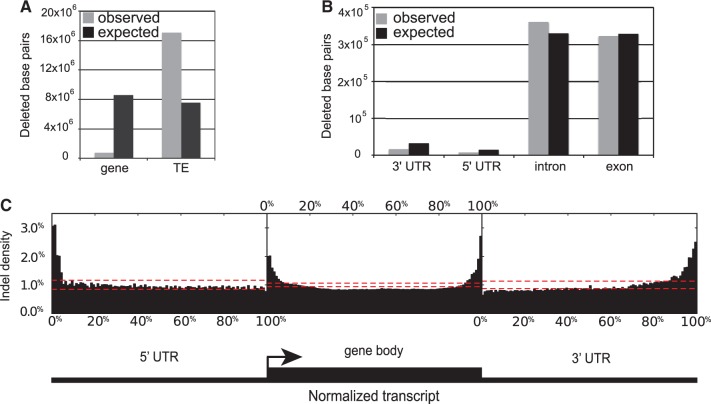
Distribution of indel polymorphisms in genes and TEs. The observed and expected number of nucleotide sites in segregating indels among (*A*) genes and TEs, and (*B*) different gene components. (*C*) Indel density along a normalized transcript, using data from all annotated genes overlapping with segregating indels. Each genic region (5′ UTR, gene body consisting of exons plus introns, and 3′ UTR) was divided into 100 equally sized bins, and for each bin the relative density among all polymorphic indels was recorded (*y*-axis). The distribution of bin densities is expected to approximately follow a binomial distribution. The red dashed lines indicate the upper and lower 95% confidence bounds for a binomial distribution with *P* = 0.01 and *n* given by the total number of inserted/deleted base pairs observed in that region.

Indels were assigned as genic if the indel interval intersected with an annotated gene, including both coding and noncoding (introns) gene components. The same was done for annotated TEs. We find a strong enrichment of indels in TEs and corresponding paucity of indels in genes. The density of indels in TEs is 244.8 indels per Mb of TE sequence. Collectively, these account for 79.3% of all observed indel nucleotides, as compared with 35.1% expected under the null model. In contrast, there are 48.2 indel per Mb of genic sequence, accounting for only 3.5% of all observed indel nucleotides, as compared with 39.9% expected under the null model (fig. 3*A*). At the nucleotide level, the nonrandom distribution among these genomic categories is highly significant (*P* < 2.2 × 10^−^^16^ in a χ^2^ test for independence). These results suggest that there is strong purifying selection against the majority of large indels that arise within genes.

By comparison, 27.4% of all observed SNPs are in coding regions (table 2), nearly 8-fold higher than the corresponding proportion for large indels. The SNPs within coding sequences are predominantly synonymous (table 2), but even nonsynonymous SNPs comprise a higher proportion of all SNPs (8.0%) than the proportion of indel nucleotides in genes (3.5%). Under the assumption that SNPs and large indel mutations occur at roughly the same frequency throughout the genome, this result would suggest that the average large indel in a gene experiences stronger purifying selection than the average nonsynonymous mutation. By contrast, large indels in TEs appear to be under weaker purifying selection than synonymous SNPs.

Despite the fact that only a small fraction of the observed indels occur within genes, we do find 414 indels disrupting 598 genes among the nine nonreference accessions. Comparing between the resequenced inbred lines, we find an average pairwise difference of 204 indel containing genes, or approximately 0.7% of all annotated genes. Assuming deletion alleles for these genes are predominantly nonfunctional and recessive, we would expect that they could be complemented in a hybrid containing a functional allele. That is, if we made synthetic hybrids between any of the nonreference Iron Mountain inbred lines sampled here we would expect the average hybrid to mask 204 nonfunctional recessive genes contributed by its parents.

We were interested to see what types of genes are affected by indels. Using *M. guttatus* paralogous gene family clusters from Phytozome (www.phytozome.net, last accessed date December 27, 2013; version 8.0), we found that genome-wide the median cluster includes six genes. For genes with a large indel, the median cluster size is 21 genes. This difference is significant (Mann–Whitney *U* test *P* < 2 × 10^−^^16^) and provides evidence for enrichment of indels among large gene families. This finding is consistent with the hypothesis that selection is weaker against indels in large gene families with many redundant paralogs when compared with small gene families (MacArthur et al. 2012), but may also reflect an elevated indel mutation rate through illegitimate recombination among paralogs. The most affected gene families include a putative nucleotide-binding site leucine-rich repeat (NBS–LRR) family (Phytozome v8.0 gene family no. 31803493), two putative F-box domain families (Phytozome v8.0 gene family no. 31838851 and no. 31808429), and a putative cytochrome P450 family (Phytozome v8.0 gene family no. 31803960).

To understand where large indels tend to occur in genes, we determined their distribution across various genic components. The indels are slightly, albeit significantly, enriched in introns and underrepresented in untranslated regions (UTRs) and exons (all χ^2^ goodness-of-fit *P* < 0.001; fig. 3*B*). We also looked for spatial patterns at finer resolution. To accomplish this, we first partitioned all indel-containing genes into three functional components, the 3′ and 5′ UTRs and the gene body (exons and introns), and then within each component normalized to a standard length. For both UTRs, there is significant enrichment of indels distal to the coding portion of the gene (fig. 3*C*). Within the gene body, there is significant enrichment on the 5′ and 3′ extremities. These results suggest that indels tend to accumulate near the periphery of all three genic components. A similar result has been seen for small indels (<60 bp) in humans (Montgomery et al. 2013). These patterns may reflect weaker selection against indels affecting peripheral components of genes when compared with more direct hits.

We then looked at the 1,855 (44.8%) large indels that are at least 90% constituted by a single annotated TE. These indels are likely associated with the mobility of a single element, and can be used to estimate the relative activity of various TE classes (supplementary table S2, Supplementary Material online). The most abundant class of TEs associated with large indels are MULEs, which account for 464 indels (25% of all indels constituted by a single annotated TE). This is an enrichment of approximately 1.6-fold relative to the frequency of MULEs among annotated TEs in the reference genome (Fisher’s exact test *P* = 1 × 10^−^^8^). On the other end of this spectrum are Gypsy and helitron elements. Each shows 1.4-fold reduction relative to expectation (Fisher’s exact test *P* = 3.8 × 10^−^^6^ and 9.5 × 10^−^^7^, respectively). Following Bonferroni correction, the families with significantly higher than expected activity are MULE and TRIM, while those families with significantly lower than expected activity are helitron, Gypsy, and LARD (supplementary table S2, Supplementary Material online). Among the eight most overrepresented TE families, seven are class II cut-and-paste DNA transposons, while all but one of the five class I copy-and-paste retrotransposon families were represented at expected levels or significantly underrepresented. We note that our indel discovery strategy allows the detection of novel insertions alleles only if they include the reference line, or deletion alleles among any combinations of the other nine nonreference lines. This creates a bias in favor of finding deletions relative to insertions, which in turns favors the discovery of polymorphic class II DNA transposons. This bias likely plays a role in differentiating the activity of class I from class II TEs. That said, there remain considerable differences among elements of the same class, and these contrasts should be unaffected by the discovery bias noted earlier.

### Strength of Selection on Large Indels

Alleles under positive or negative selection are expected to be on average younger than neutral alleles found at the same frequency in a population (Maruyama 1974). Using a diffusion approximation that assumes no dominance and a constant population size, Maruyama (1974) showed that the expected mean allele age is symmetric for positive and negative selection coefficients of the same magnitude. Kiezun et al. (2013) extended this result to create an estimate of the strength of selection on different types of rare mutations using the relationship between intra-allelic nucleotide diversity (π_A_) and mean allelic age. We apply this method to our data to estimate the strength of selection on the subset of large indels within genes relative to synonymous and nonsynonymous mutations. To do this, we first partition the three mutation types based on their observed allele frequency in the sample population. Then, conditioning on alleles at the same frequency, we estimated π_A_ in a 500 bp haplotype centered on the focal mutation. We chose this small haplotype size to minimize the probability that our intra-allelic sample has experienced a recombination event.

For all synonymous mutations across all allele frequencies, the grand mean π_A_ was 0.0087, which can be interpreted as the expected diversity accumulated over the average coalescent time for a neutral allele in the population (i.e., after 2*N*_e_ generations). Mean π_A_ for genic deletion alleles, nonsynonymous mutations, and synonymous mutations at 20% allele frequency are 0.0031, 0.0047, and 0.0055, respectively. All values are lower than the grand mean π_A_, which is expected because conditioning on alleles at 20% frequency should bias the pool toward younger—and hence less diverse—intra-allelic haplotypes. By dividing by the grand mean π_A_ for all synonymous mutations, mean π_A_ for alleles at 20% frequency can be converted to average coalescent times—expressed in 2*N*_e_ generations—of 0.36, 0.54, and 0.63 for genic deletion alleles, nonsynonymous mutations, and synonymous mutations, respectively (supplementary fig. S6, Supplementary Material online). The lower values observed for genic deletions and nonsynonymous mutations imply younger alleles and stronger selection on average for these loci when compared with putatively neutral synonymous loci, and that the difference from neutrality is greater for genic deletions than for nonsynonymous mutations.

With these coalescent time estimates, we can approximate the magnitude of the average coefficient of selection on each mutational class using a diffusion approximation (Kiezun et al. 2013). The magnitude of the mean population-scaled coefficient of selection ($\bar{N_{e}S}$) on synonymous mutations at a 20% allele frequency is estimated to be approximately |1.9 $\bar{N_{e}S}$|, while nonsynonymous mutations are |2.7 $\bar{N_{e}S}$|, and genic deletions are |5.4 $\bar{N_{e}S}$| (supplementary fig. S6, Supplementary Material online). For alleles at a 20% frequency, genic deletions are the youngest class and are estimated to experience 2.8 times stronger selection than synonymous mutations. Neutral alleles are expected to be ≤ |1 $\bar{N_{e}S}$|, and thus all loci at a 20% frequency are younger (i.e., harbor on average lower π_A_) than would be expected under neutrality, even synonymous polymorphisms. Because the expected allele age is symmetric for positive and negative selection coefficients of the same magnitude (Maruyama 1974), we cannot conclude that genic deletions experience negative selection. However, when coupled with their low average MAF (table 2) and scarcity relative to their mutational target size (fig. 3*A*), it seems likely that genic deletion alleles at 20% allele frequency acquire large absolute values of $\bar{N_{e}S}$ primarily through negative purifying selection.

### Identification of Putatively Positively Selected Indel Alleles

**Fig. 4.—:**
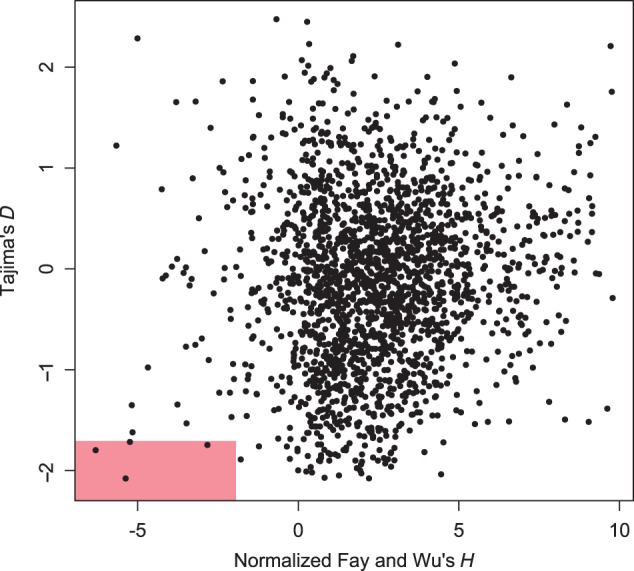
Identification of indel loci putatively under positive selection. The plot includes Normalized Fay and Wu’s *H* and Tajima’s *D* estimates for each indel. The red box in the lower left corner indicates the area outside the lower 95% confidence interval of both metrics as assessed by coalescent simulation.

Despite an expectation of neutral or negative purifying selection on large indels most of the time, a new indel allele may on occasion become the target of positive selection. Several recent studies have linked adaptive traits to novel TE insertion events (Aminetzach et al. 2005; Studer et al. 2011). To identify potential targets of positive selection from among the set of indels we discovered, we first polarized all indels using our outgroup accessions and then extracted a 10 kb segment centered on the indel locus for each ingroup accession. We then applied Tajima’s *D* and Normalized Fay and Wu’s *H* (Zeng et al. 2006) to these sequences. Four derived insertion alleles—ranging from 2,140 to 5,701 bp—showed strongly negative values for both tests, which is an indication of recent positive selection (fig. 4). In addition, all four insertions alleles are found at ≥80% frequency in the focal Iron Mountain population, also suggestive of positive selection. Three of the insertion alleles are near the upstream region of a gene (2,056, 3,981, and 13,701 bp from the start codon), whereas the fourth allele is 3,211 bases downstream of the nearest gene’s stop codon. None of these insertions are clearly annotated as a single TE, though two intersect with more than one TE. The associated genes are annotated as an F-box gene (mgv1a018496m), a thaumatin family gene (mgv1a019837m), an eIF-3 family gene (mgv1a012800m), and a mitogen-activated protein kinase (mgv1a003728m). It is possible that the signatures of positive selection detected are actually associated with other polymorphisms in or near these genes. Alternatively, by analogy to the expression changes in the maize *tb1* gene driven by a large upstream insertion thought to have been selected under domestication (Studer et al. 2011), these indels may themselves be driving expression changes that are under positive selection.

## Discussion

Several recent studies have revealed a wealth of large polymorphic indels at a species-wide level (Emerson et al. 2008; Conrad et al. 2010; Cao et al. 2011; Long et al. 2013). Here, we extend these findings to a single population of *M. guttatus* originating from an alpine population on Iron Mountain, OR. These findings complement the relatively small number of other population-level studies that have been done to date (Cridland and Thornton 2010; Mackay et al. 2012; Feulner et al. 2013; Montgomery et al. 2013), and to our knowledge mark the first study of this type in plants.

In total we find 4,142 distinct indel events segregating among ten inbred accessions extracted from Iron Mountain*.* On average, each accession bears 1,422.6 deletions totaling 6.6 Mb relative to the reference genome. In contrast, each accession differs on average from the reference genome at only 0.42 million SNPs. Thus, there are 16 times as many nucleotides affected by indels as by SNPs in the average accession.

Assuming that large indel polymorphisms in this population are near mutation-selection balance, estimates of population genetic metrics can give us insight into the evolutionary forces involved. We find that indel size and position vis-à-vis genes are both strong predictors of allele frequency (fig. 1 and table 2), with larger indels and genic indels tending to have rarer minor alleles. We also find that 71.8% of the minor alleles are derived in the Iron Mountain population (table 2), a value that is significantly higher than what was found for all classes of SNPs. Several of our findings mirror those from a recent study by Long et al. (2013). They sampled 180 *Arabidopsis thaliana* lines from Sweden and found that large indels alter megabases of genomic content between lines, including estimates of 200–300 present/absent genes. They also discovered that indels are a bit more likely to have a rare derived minor allele when compared with SNPs, and that this pattern intensifies when looking only at genes. Finally, Long et al. (2013) also found a positive relationship between the abundance of indels and SNPs in the genome.

We estimate that the average coefficient of selection for genic indel alleles is approximately 2-fold stronger than selection on nonsynonymous mutations and approximately 3-fold stronger than synonymous mutations when conditioning on alleles at a 20% frequency (the rarest class of alleles from which we can calculate π_A_). These results are consistent with previous studies on the population genetics of large indels in *Drosophila* (Emerson et al. 2008; Cridland and Thornton 2010; Mackay et al. 2012).

Within this pool of variation, we also find a small number of young deletion alleles that may be under positive selection. By combining two tests for positive selection, we identify four indels as outliers (fig. 4), three of which involve novel high-frequency derived insertion alleles near the 5′ start site of a gene. Without additional experimental evidence, we cannot yet say whether these indels affect the regulation of the adjacent genes, have another direct effect on fitness, or are not themselves targets of selection but subject to hitchhiking from selection on linked sites. Nevertheless, the approach we outline here highlights a practical genomic scan that could be used to identify candidate regulatory polymorphisms that are visible to selection.

In terms of the genes affected, the most dramatic finding is that 71 of the 598 genes segregating for a polymorphic indel belong to the NBS–LRR family, a value that is greater than twice the random expectation based on the NBS–LRR gene family size (Fisher’s exact test *P* = 1.5 × 10^−^^10^). We believe that this is not simply an artifact of alignment mapping errors in the highly repetitive NBS–LRR family because we see nonsignificant enrichment within other large gene families that have even higher levels of similarity among paralogs (results not shown). Interestingly, NBS–LRR genes have also been found to be enriched for large indels in soybean and *Arabidopsis* (Shen et al. 2006; McHale et al. 2012), suggesting that they are common targets for indel mutations among plants. There are several possible explanations. The NBS–LRRs gene family is thought to have an unusually high rate of gene copy turnover (Baumgarten et al. 2003; Meyers et al. 2005). This creates many highly similar paralogs, which in turn increases the possibility that some NBS–LRR members may serve redundant functions, and therefore their loss may be selectively neutral. Similarly, Gos and Wright (2008) have suggested that NBS–LRRs genes can be functionally neutral in the absence of the pathogen they detect, and under these circumstances their loss or inactivation would be selectively neutral with respect to selection. In addition to these neutral explanations, research has also shown that the maintenance of some NBS–LRRs can come at a cost. Bomblies et al. (2007) found that some NBS–LRR genes play a role in hybrid necrosis, which results when a NBS–LRR incorrectly sets off plant defense pathways in a hybrid background due to off-target stimulation. This form of hybrid mortality could pose a significant fitness cost in a highly outcrossing species like *M. guttatus* and could select for nonfunctional indel alleles among offending NBS–LRRs during times of population admixture. Also, rather than simply being functionally neutral, at least one NBS–LRRs has been shown to decrease fitness in the absence of the pathogen it recognizes (Tian et al. 2003). Any of these scenarios could lead to balancing selection among functional and a nonfunctional NBS–LRR alleles, and may in part explain why NBS–LRRs are highly enriched for polymorphic indel mutations.

Methods for identifying SVs will improve rapidly as long-read sequencing matures, and we anticipate great interest in their discovery and evolution. Here we find thousands of indel polymorphisms using short-read sequencing, and by employing a number of filters, achieve a 72% validation rate for our in silico predicted indels. Though we found it difficult to generate a high-confidence list of other SVs (inversions and transpositions), several of the filters we describe can be fruitfully used in future studies using either short- or long-reads.

## Supplementary Material

Supplementary tables S1–S3 and figures S1–S6 are available at *Genome Biology and Evolution* online (http://www.gbe.oxfordjournals.org/).

Supplementary Data
