# Supplementary material for: The Standing Pool of Genomic Structural Variation in a Natural Population of Mimulus guttatus
Source: Genome Biol Evol. 2013 Dec 12;6(1):53–64. doi: 10.1093/gbe/evt199 (PMC3914686; doi:10.1093/gbe/evt199)
Supplement: Supplementary Data [file supp_6_1_53__index.html]

The standing pool of genomic structural variation in a natural population of Mimulus guttatus — The Standing Pool of Genomic Structural Variation in a Natural Population of Mimulus guttatus — Supplementary Data 

# The Standing Pool of Genomic Structural Variation in a Natural Population of *Mimulus guttatus*

## Supplementary Data

files

**Files in this Data Supplement:**

- Supplementary Data - docx file
- Supplementary Data - xls file
- Supplementary Data - xls file
- Supplementary Data - xls file
