# Supplementary material for: The Standing Pool of Genomic Structural Variation in a Natural Population of Mimulus guttatus
Source: Genome Biol Evol. 2013 Dec 12;6(1):53–64. doi: 10.1093/gbe/evt199 (PMC3914686; doi:10.1093/gbe/evt199)
Supplement: Supplementary Data [file supp_evt199_SUPPLEMENTARY_INFORMATION.docx]

**SUPPLEMENTARY INFORMATION**

**Figure S1: Alignment of read pairs along *Mimulus* chromosome 9.** The two dot-plots show the left and right read pair positions for high-quality paired read alignments (BWA mapping quality ≥ 29) in the reference line (IM62; left) and a non-reference line (IM693; right). Paired reads that align to the reference genome in the expected location fall along the diagonal, while all abnormally aligned reads are represented above the diagonal. Concentrations of abnormal read pairs in IM62 indicate regions in which read pair data from other lines would be unreliable for detection of SVs. The abundance of abnormally aligned read pairs indicates the extent of the technical challenge that must be overcome to filter the signal from the noise.


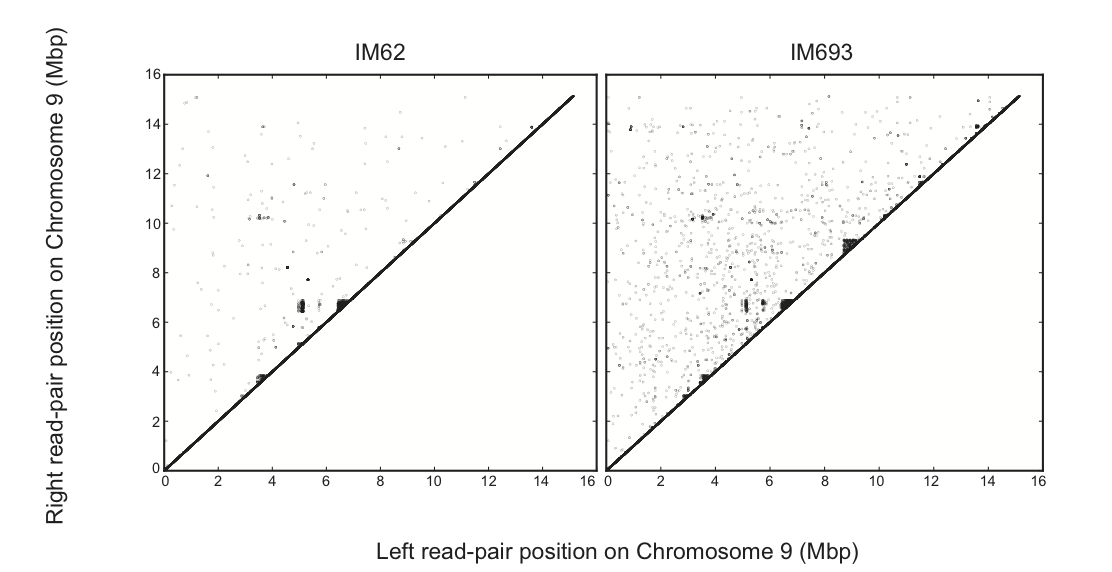


**Figure S2: Nucleotide diversity (*π*) within putative inversion intervals for both the inverted and the reference genome form of an inversion.** The black line shows the pattern expected if nucleotide diversity within putative inversion haplotype does not differ between inversion haplotypes. The seven inversion events (red points) showing unusually low nucleotide diversity among inverted haplotypes were our targets for validation. We chose these with the rationale that the rare form of the inversion might be recent and have little nucleotide diversity, and that this signature would be unlikely to come from collinear regions, thus enriching these seven for *bona fide* inversions. However, we could not generate unique primer pairs for any of these seven.

**Figure S3: Deletion allele frequency estimates from deep population sampling (*x*-axis) and whole genome resequencing of ten inbred lines (*y*-axis).** The linear regression is shown in red with the corresponding *R^2^*.

**Figure S4: Relationship of deletion frequency to coverage and nucleotide divergence.** Left: there is only a weak negative relationship between the number of read pairs aligned and the number of deletions found within each of the nine non-reference lines. Right: there is a positive relationship between the pairwise nucleotide divergence and the number of deletions found between IM62 and each of the nine non-reference lines. Linear regressions are shown in red with corresponding *R^2^* values.

**Figure S5: Distribution of genes, repeats, centromeres, alignable regions, and indels along *M. guttatus* chromosome 1.** Chromosome 1 has been broken into 25 kb segments, and for each segment various proportions or counts are represented by an associated legend to the right of each subplot. Chromosome 1 appears to be acrocentric, with genes clustered primarily on the left side and the centromeric/repetitive fraction dominating the right side of the. The “Proportion Alignable” refers to the proportion of sites in a 25 kb segment that support at least one aligned paired-end read with a mapping quality scores ≥ 29. As expected, the gene rich left side of the chromosome is enriched for alignable sites, and as a consequence is expected to be more available for indel discovery. The other 13 *M. guttatus* chromosomes (not shown) have similar patterns. Centromeric repeats were annotated by blast search using the *M. guttatus* 728 bp centromeric repeat sequence ([Fishman and Saunders 2008](#_ENREF_17)).

**Figure S6: Estimating the coefficient of selection**

(A) Table of all intra-allelic nucleotide diversity results from 500 bp haplotypes surrounding genic deletions, nonsynonymous and synonymous mutations for alleles at 20% to 90% frequency. (B) Theoretical relationship between strength of selection and mean allelic age for alleles at 20% frequency.

**Table S1: Validation results.** The table includes the genomic location of each targeted indel, the primer pairs used for PCR and validation results.

**Table S2: Indels associated with transposable element families.**  The first worksheet reports the transposable element results aggregated to the level of families of related elements, while the second includes all results individualized to specific elements sub-families found within *M. guttatus.* For each TE counts are given for the 2 × 2 contingency table used to contrast observed polymorphisms versus expected on the basis of genomic abundance. In addition results from the Fisher’s Exact Test, the odds ratio, and observed fold change are given.

**Table S3: Observed proportions of various configurations of bitwise SAM flags.** For each pair of bitwise SAM flags the putative structural interpretation is given along with its frequency among the all reads for each resequenced line.
